# Farming experience, personal characteristics, and entrepreneurial decisions of urban residents: Empirical evidence from China

**DOI:** 10.3389/fpsyg.2022.859936

**Published:** 2022-07-29

**Authors:** Deshui Zhou, Lele Li

**Affiliations:** ^1^School of Finance and Public Management, Anhui University of Finance & Economics, Bengbu, China; ^2^School of Labor and Human Resources, Renmin University of China, Beijing, China

**Keywords:** farming experience, entrepreneurial decision, personal characteristics, physical capital, urban residents

## Abstract

Entrepreneurship is an important way to provide solutions for social employment problems. Using data from the 2016 China Labor Force Dynamic Survey (CLDS), we explore the influence of farming experience on urban residents’ entrepreneurial decisions at the theoretical and empirical levels. A Probit model with instrumental variables method was used to analyze the influence of farming experience on urban residents’ entrepreneurial decisions, while a mediating effect model was used to test its channels of action. The results show that: (1) farming experience can contribute to the entrepreneurial decision of urban residents relative to those without experience in farming. To overcome possible endogeneity issues, an Eprobit model based on the estimation of instrumental variables was used for testing. (2) Heterogeneity tests based on age, city type, and physical capital found that this effect was more significant in urban residents with non-capital cities, middle-aged groups, and high-material capital. (3) Farming experience indirectly drives entrepreneurial decisions through the mediating role of promoting positive personality traits, such as “optimism” and “mutual aid consciousness.” Therefore, the farming experience has a positive effect on urban residents’ entrepreneurial decisions and helps to understand the deeper influence of micro-individual characteristics on entrepreneurial decisions in the urbanization process.

## Introduction

Promoting employment through entrepreneurship is an important element of China’s employment priority strategy. By the end of 2020, the number of market entities of all kinds in China had grown to 144 million, among which the number of self-employment as a direct business activity had reached 967 million (National Bureau of Statistics, 2021). Self-employment is not only a specific extension of “entrepreneurship,” but also an employment choice made by entrepreneurs according to the economic market environment (Colovic and Schruoffeneger, 2021), which is conducive to gaining social respect, realizing self-worth, and improving the level of economic returns (Blundel et al., 2010). Especially under the influence of the overall downward trend of the world economy, entrepreneurship-led employment has the important task of improving people’s livelihood and promoting social development, and has become one of the driving forces for achieving macroeconomic growth (Qin and Kong, 2021; Azoulay et al., 2022). Under the influence of the epidemic, people’s employment and entrepreneurship are facing severe challenges (Dias et al., 2022). Supporting and encouraging different groups to engage in entrepreneurship has become a public policy direction of concern for the Chinese government (Bublitz et al., 2020).

Therefore, it is important to study and explore the driving factors of entrepreneurship. In terms of the driving factors influencing individual entrepreneurial choices, the accumulation of knowledge, skills, cognition, and capital involved in prior experiences play an important role in facilitating the choice of entrepreneurial behavior (Hockerts, 2017). Also, farm-related experience is an important micro factor in the structure of the rural economy and a typical feature of the rapid urbanization process in China (Zhong and Chen, 2014). This process has resulted in a large number of rural laborers moving to urban areas or transforming into urban populations, who are divorced from agricultural labor and engaged in secondary or tertiary non-farm employment (Zhao et al., 2022). In the process of non-farm employment, the role of farming experience is reflected in at least three aspects: (1) it provides a comparison between the benefits of farming and non-farm employment and provides a reference for their subsequent career choices (Zhong and Chen, 2014). (2) Farming experience is a survival skill that expands workers’ human capital accumulation. (3) It helps workers adapt to different labor styles in different environments and enhances their urban. At the same time, it is conducive to workers’ adaptation to different environments and enhances their urban adaptation ability. As an important part of workers’ prior experience, past experiences such as farming experience may have long-term effects on individual behavior (Kendler et al., 2002).

Previous studies have laid a good foundation for this study. Some scholars have conducted in-depth research on the impact of bad experience on individual economic behavior. For example, some scholars pointed out that farmers who experienced famine in their early years tend to have a higher tendency to save (Cheng and Zhang, 2011), and individuals with a difficult childhood experience are less likely to engage in self-employment (Drennan et al., 2005). Managers of firms who have had early experiences of famine will be less effective in leading the firm to make investments (Donaldson, 1990; Malmendier et al., 2011). In addition, urban residents with a rural upbringing experience will have a lower probability of participating in the stock market (Jiang et al., 2018). Fan (2017) states that people with upbringing experience are more inclined to purchase insurance and reduce their risky asset holdings.

However, some scholars have also identified individual-level traits and possession of experiences as important factors driving entrepreneurial behavioral choices (Clarysse et al., 2011; Iversen et al., 2016). Prior life experiences facilitate the accumulation of human capital, enhance information intake and interpretation, and facilitate entrepreneurial opportunity identification (Shane, 2000; Rerup, 2005). The relationship between individuals having relevant work experience and entrepreneurship shows an inverted U-type relationship that rises and then falls, but the positive effect remains significant (Rider et al., 2013). Similar to rural-related experience, a person’s prior relevant experience has a significant driving effect on their decision to engage in venture-based entrepreneurship (Kendler et al., 2002). It has also been argued that corporate executives with early adverse experiences significantly increase social giving and treat others more generously (Yuan et al., 2019). A study based on data from the China Family Panel Studies (CFPS) concluded that the experience of going to the countryside could significantly increase the probability and size of investment in stocks and financial assets of urban households (Zhou et al., 2020).

Based on the existing research, the interest and question of this paper are to explore how the farming experience affects entrepreneurial decision? Further, if there is a significant effect, through which channels does it affect the entrepreneurial decision of the population? Does the identification of this effect vary across groups? Clarifying these questions will be useful in promoting the quality of employment and helping to promote the employment-first strategy.

The marginal contributions of this paper are as follows: First, from the perspective of farming experience, this paper excavates the influencing factors of urban residents’ entrepreneurship, enriching the research field of people’s entrepreneurial behavior. Second, it puts forward the theoretical hypothesis that agricultural experience affects entrepreneurship and empirically tests the indirect effect of personality characteristics in the process of agricultural experience affecting entrepreneurship, which expands the research depth. Third, the group regression is carried out from the heterogeneity of city type, age stage, and material capital, trying to capture which groups of farming experience are more likely to affect entrepreneurial activities.

## Literature review and research hypothesis

Since China’s reform and opening up in 1978, the development of urbanization and industrialization has promoted the rapid growth of China’s economy (Duan and Zhang, 2009). However, constrained by the dualistic system of urban–rural division, the transfer of agricultural labor during urbanization, although meaningful for promoting employment, has not been effectively linked to urban public services and management associated with the household registration system (Yu and Gao, 2009). This means that labels associated with rural areas, such as agricultural household registration and migrant workers, tend to impede their mobility to higher levels of the labor market (Wu and Zheng, 2018). With the rapid advancement of China’s household registration system reform and the citizenship process of the agricultural population, new citizens have gradually become the driving force influencing urban economic development (Liang et al., 2022), and the proportion of urban residents with experience in farming has gradually increased. If urban residents have engaged in agricultural labor, it may have a two-way effect of promoting and inhibiting residents’ entrepreneurship. On the one hand, past experience can enhance one’s experience and personality traits, and give one the ability to cope with corresponding events and risks (Hoff and Stiglitz, 2016). At the same time, it is conducive to accumulating a certain amount of “potential human capital,” helping one to cope with setbacks and improve self-adaptability, and may have some positive effects on one’s long-term development (Chetty et al., 2010). On the other hand, the farming experience tends to be constrained by the rural environment and has a solidifying effect on their cognitive thinking and behavioral performance, which may reduce their preference for venture capital and have a negative effect on their personal and economic behavior (Jiang et al., 2018). Therefore, the theoretical impact of farming experience on entrepreneurial decisions is uncertain and needs to be tested empirically.

Although farming experience may have a significant effect on entrepreneurial decisions, this hypothesis may have heterogeneous effects on different groups. First, for different age groups, middle-aged groups are more likely to have farming experience, while youth groups are largely disconnected from land in terms of income (Wang, 2008). Therefore, the impact of farming experience on economic activity may be higher for middle-aged groups than youth groups. Second, for different city levels, provincial capitals are more economically developed, with larger cities and earlier urbanization (Zhu and Yang, 2018), and thus policy support and economic advantages may form a substitution effect on individual factors. For the vast majority of non-capital cities, which are generally at the stage of rapid urbanization and have a greater hierarchy of economic development, the past experiences possessed by the labor force may be more sensitive to the impact of socioeconomic activities and generate a relatively larger marginal contribution compared to the provincial capitals. Third, for groups with different physical capital, it is easier to obtain desirable income returns in the labor market among high-physical capital groups, whose past experiences may be more effective in driving current economic activities (Shum and Faig, 2006), which is conducive to give better play to the advantages of their previous experience and have an impact on economic behavior.

Traditionally, agricultural labor is the main source of income for rural laborers and is responsible for maintaining household consumption and savings. As a result, rural laborers have to bear the risks involved in farming. In addition, if natural or man-made disasters occur, they may lose their source of income and fall into a “livelihood crisis” (Crupi et al., 2022). Because farming is subject to a variety of factors such as natural environment, weather conditions, and environmental changes, its output is far more uncertain than that of other industries (Timmer, 1988), and only hard-working and intelligent workers can earn a good income (Bernstein, 2001). So the complexity involved in the farming experience may be conducive to shaping an optimistic personality and the accumulation of the ability to cope with such “crises.” The sense of mutual help is likewise another important aspect in shaping personality traits. The sense of mutual help is inherited, and the promotion of traditional Chinese virtues is more deeply reflected in the values at the individual level. As an extension of personal traits, it not only helps to regulate the framework of economic behavior for those involved in economic activities, but also helps to stimulate various potentials of individuals in economic activities and increase their enthusiasm in economic activities, providing vitality for the development of market economy (Luthans et al., 2007).

Entrepreneurs are not sure if they are suitable to start a business at first, but they can learn from their existing experiences and become optimistic and confident as they gain social experience (Fraser and Greene, 2006). Choosing to start a business and becoming an entrepreneur are two processes of learning, and the personality characteristics of entrepreneurs have an important impact on the learning process of entrepreneurs (Littunen, 2013). Personality traits as a unique resource endowment of entrepreneurs are conducive to entrepreneurial performance (Zhang and Bruning, 2011), and personality traits remain largely stable over the life cycle after being formed at an early age (Caspi et al., 1984). Studies have found that personality quality with resilience as the core significantly improves entrepreneurial performance (Hmieleski and Carr, 2009). At the same time, entrepreneurship is a difficult process, which requires entrepreneurs to have comprehensive abilities (Cubero et al., 2022); among them, the excellent traits that entrepreneurs possess are crucial in the entrepreneurial process (Aldrich and Cliff, 2003), which helps to provide entrepreneurs with high motivation in the face of adversity and enhances the success rate of entrepreneurship (Urban et al., 2022).

Based on the above analysis, this paper puts forward the following three hypotheses:

**Hypothesis 1: Farming experience has a significant impact on urban residents’ entrepreneurial decisions.**

**Hypothesis 2: The effect of farming experience on urban residents’ entrepreneurial decisions is more significant in individuals with middle-aged groups, non-capital cities, and high-physical capital.**

**Hypothesis 3: Farming experience is conducive to positive personality traits and indirectly contributes to entrepreneurial decisions.**

## Materials and methods

### Data description

The data in this paper are from the 2016 China Labor Force Dynamic Survey (CLDS) released by the Social Science Survey Center of Sun Yat-sen University. The survey is extensive, covering many aspects of the labor force, such as occupation, health, education, mobility, family, and community. In the survey process, the multi-stage, multi-level probability sampling method proportional to the labor force scale is adopted to ensure the randomness and unbiased of the data. This survey is widely represented in China, covering 29 provinces, autonomous regions, and cities (excluding Hong Kong, Macao, Taiwan, Hainan, and Tibet); it uses people aged 15–64 as respondents and adopts a multi-stage, multi-level, and probability sampling method to conduct a systematic survey. Moreover, we selected the sample of 18–59-years-old registered residence in urban areas as the research object. Because more rural labor force continues to work from affairs to agriculture, we cannot select the samples from the samples that do not work in business agriculture but work in agriculture during the survey. In this way, it can test the probability and behavior choice of the group with agricultural experience among urban residents compared with the sample without agricultural experience. If all samples are included in the research scope, there will be a certain estimation deviation, and then it is impossible to accurately estimate the actual impact of farming experience on the entrepreneurial decision. Urban residents are basically separated from agricultural labor and can accurately estimate the actual impact of agricultural experience on their entrepreneurial decision. After data screening and eliminating the unqualified samples, the total number of samples is 2,690.

### Variable selection

The explanatory variable in this paper is Entrepreneurial Decision. The corresponding design of the questionnaire is “have you tried to start a business.” We assign the corresponding answer of “yes” to 1 and the corresponding answer of “no” to 0. The proportion of the sample that has tried to start a business is 9.54%. The explanatory variable in this paper is Farming Experience, and the corresponding design in the questionnaire is “Have you ever worked in farming.” We assign the answer “yes” to 1 and the answer “no” to 0. The proportion of urban residents with farming experience in the sample is 22.98%. It should be noted that Table 1 is a descriptive analysis of explanatory variables and explained variables and examines the correlation between single variables. The total samples controlled in this paper are based on the benchmark results in Table 3, that is, the sample size when all variables are controlled. This includes the case that there are some missing values in the control variables, so these missing values are eliminated in the regression process, resulting in inconsistency in the number of samples.

**TABLE 1:** Comparison of entrepreneurial means.

Projects	Mean	Sd	Obs.
Experience in farming	0.1363	0.3434	675
No farming experience	0.0934	0.2910	2270
Difference in sample means between two types of sample startups	0.0429		

To visually determine the difference between the means of the two types of samples of urban residents with and without experience in farming who have engaged in entrepreneurial decisions, the two groups of samples are compared in Table 1. It can be seen that in the subsample with farming experience, the percentage of those who have engaged in entrepreneurship is 13.63%, while the percentage of those who have engaged in entrepreneurship in the subsample without farming experience is 9.34%, and the difference in the percentage of entrepreneurial decisions between the two types of samples is 4.29%. This indicates that intuitively and statistically residents with farming experience have a higher proportion of entrepreneurship, but the causal relationship between the farming experience and an entrepreneurial decision needs to be further tested empirically.

For control variables, the effects of “Personal Characteristics,” “Family Characteristics,” “Socio-economic Characteristics,” and “Regional Dummy Variables” were examined separately. For “Personal Characteristics,” the mean value of Gender is 0.452, indicating that the overall ratio of male to female respondents is close to balanced. The mean value of age is 41.74 years old, indicating that the respondent sample is dominated by the middle-aged labor force. The proportion of the sample with Junior High School, High School, and University and above is 26.8, 28.1, and 30.8%, respectively, indicating that the sample is biased toward the middle to upper education level. The mean value of marriage is 0.79, indicating that the majority of the respondent sample is married. The proportion of respondents with a drinking history was 19.1%, indicating a low proportion of the sample with a history of alcohol consumption. For “Family Characteristics,” it controls Family Medical Expenses, Household Housing Expenditure, and Land Acquisition. For “Socio-economic Characteristics,” it investigates Housing Provident Fund, Internet Banking, Income Satisfaction, and Social Donation. For “Regional Dummy Variables,” taking the middle as the reference group, the East and West dummy variables are included as the control variables. Variable definitions and descriptive statistics are shown in Table 2.

**TABLE 2:** Statistical description of variables.

	Variable definition	Mean	SD
Entrepreneurial decision	Yes = 1, No = 0	0.0954	0.294
Farming experience	Yes = 1, No = 0	0.2298	0.421
**Personal characteristics**			
Gender	Male = 1, Female = 0	0.452	0.498
Age	Continuous variable of age	41.74	11.37
**Education level (reference: below primary school)**			
Junior high school	Junior high school is 1, otherwise is 0	0.268	0.443
High school	High school is 1, otherwise is 0	0.281	0.449
University and above	University and above is 1, otherwise is 0	0.308	0.462
Marriage	Married is 1, otherwise is 0	0.790	0.407
Drinking history	Yes = 1, No = 0	0.191	0.393
**Family characteristics**			
Family size	Number of Families	3.616	1.733
Family medical expenses (log)	Continuous variables of family medical expenditure	6.529	3.658
Household housing expenditure (log)	Continuous variables of household housing expenditure	7.835	1.103
Land acquisition	Family land expropriated is 1, otherwise is 0	0.0069	0.0832
**Socio-economic characteristics**			
Housing provident fund	Yes = 1, No = 0	0.298	0.457
Internet banking use	Very unskilled, unskilled, average, relatively skilled, and very skilled are assigned as 1, 2, 3, 4, and 5, respectively	2.919	1.288
Income satisfaction	Very dissatisfied, relatively dissatisfied, average, relatively satisfied, and very satisfied are assigned the values of 1, 2, 3, 4 and 5	3.058	0.981
Social donation	Yes = 1, No = 0	0.365	0.481
**Regional dummy variables (control: Central)**			
East	East is 1, otherwise is 0	0.465	0.499
West	West is 1, otherwise is 0	0.270	0.444

### Variable setting and descriptive statistics

The explanatory variables in this paper are binary variables, so the Probit model is used for regression. Probit model is set as follows:


(1) $$P\left(Y=1|T_{i}\right)=\varphi \left(\beta_{0}+\beta_{1}X_{i}+\beta_{2}E_{i}+\mu_{i}\right)$$

In Equation 1, the *Y* denotes entrepreneurship, and if the urban labor force surveyed has tried to start a business, then *Y=1*. *T*_*i*_ denotes the vector of explanatory and control variables. *X*_*i*_ denotes the control variables, which specifically include “Personal Characteristics,” “Family Characteristics,” “Socio-economic Characteristics,” and “Regional Dummy Variables,” and *E*_*i*_ denotes the explanatory variable, farming experience. β_*0*_ are constants, β_*1*_ and β_*2*_ are coefficients to be estimated, μ_*i*_ denotes the random error term, and this paper assumes that the sample obeys normal distribution.

## Results

### Analysis of baseline regression results

Table 3 shows the results of the baseline regression of the effect of farming experience on urban residents’ entrepreneurial decisions. Models 1–4 control “Personal Characteristics,” “Family Characteristics,” “Socio-economic Characteristics,” and “Regional Dummy Variables,” respectively. In model 1, the Farming Experience is significantly positive at the statistical level of 1%, indicating that the agricultural experience can significantly improve the entrepreneurial probability of urban residents. In model 2, after adding “Family Characteristics” variables, the estimation of the coefficient of Farming Experience is still significantly positive. In models 3 and 4, “Socio-economic Characteristics” and “Regional Dummy Variables” are added as control variables, respectively, and the estimated coefficient value direction and significance degree are still basically the same. These results show that the Farming Experience is indeed conducive to improving the Entrepreneurial Decision probability of urban residents and verifying H1.

**TABLE 3:** Baseline regression of the effect of farming experience on urban residents’ entrepreneurial decisions.

	Model 1	Model 2	Model 3	Model 4
Farming experience	0.246***	0.232***	0.270***	0.283***
	(0.077)	(0.084)	(0.088)	(0.089)
**Personal characteristics**				
Gender	0.273***	0.301***	0.358***	0.363***
	(0.073)	(0.076)	(0.078)	(0.078)
Age	0.094***	0.101***	0.104***	0.111***
	(0.029)	(0.030)	(0.032)	(0.032)
Age2	−0.001***	−0.001***	−0.001***	−0.001***
	(0.0003)	(0.0003)	(0.0003)	(0.0003)
**Education level (reference: below primary school)**				
Junior high school	0.250*	0.352***	0.368***	0.374***
	(0.129)	(0.131)	(0.138)	(0.139)
High school	0.209*	0.272**	0.273**	0.287**
	(0.126)	(0.129)	(0.138)	(0.138)
University and above	0.0568	0.140	0.168	0.203
	(0.126)	(0.129)	(0.141)	(0.142)
Marriage	−0.174*	−0.260**	−0.225**	−0.211*
	(0.100)	(0.104)	(0.108)	(0.108)
Drinking history	0.127	0.105	0.129	0.155*
	(0.081)	(0.085)	(0.087)	(0.087)
**Family characteristics**				
Family size		0.061***	0.637**	0.663**
		(0.023)	(0.317)	(0.315)
Family medical expenses		0.019*	0.059***	0.055**
		(0.010)	(0.023)	(0.023)
Household housing expenditure		0.093***	0.018*	0.021*
		(0.031)	(0.0106)	(0.011)
Land acquisition		0.695**	0.081**	0.044
		(0.309)	(0.032)	(0.033)
**Socio-economic characteristics**				
Housing provident fund			−0.385***	−0.397***
			(0.080)	(0.081)
Income satisfaction			−0.116***	−0.123***
			(0.035)	(0.035)
Social donation			0.324***	0.312***
			(0.071)	(0.072)
Internet banking use			0.122***	0.128***
			(0.036)	(0.036)
**Regional dummy variables (control: central)**				
East				0.277***
				(0.089)
West				−0.034
				(0.102)
_cons	−3.330***	−4.677***	−4.884***	−4.891***
	(0.550)	(0.665)	(0.707)	(0.724)
Pseudo R2	0.0277	0.047	0.0816	0.0907
N Obs.	2,915	2,743	2,690	2,690

***, **, and * indicate significance at the 1, 5, and 10% statistical levels, respectively, with robustness standard errors in parentheses.

For control variables in Table 3, among the “Personal Characteristics,” the Gender is significantly positive at the 1% level, indicating that men have a higher probability of engaging in entrepreneurship. The older the age, the higher the probability of entrepreneurship, but the coefficient value of age squared is significantly negative, indicating that the effect on the impact of entrepreneurship decreases with a further increase in age. The probability of entrepreneurship is higher for those with Junior High School and High School education compared to those with Below Primary School, but there is no significant effect of the university and above. Relative to being married, those who are unmarried have a higher probability of starting a business. After adding all variables, we found that drinking history significantly facilitated Entrepreneurial Decision at the 10% level, However, drinking and alcoholism are not the same connotations. Alcoholism is excessive drinking, and the drinking history of this article is that drinking in the past is not drinking now. Drinking has the function of expanding social networks, which makes it easier to expand social networks and social capital, thus promoting entrepreneurship. Regarding “Family Characteristics,” Family Size significantly promoted entrepreneurship. The higher the Family Medical Expenses, the higher the probability of entrepreneurship. The higher the Household Housing Expenditure, the higher the probability of entrepreneurship. The Land Acquisition has a significant positive effect in Model 2 and Model 3, but is not statistically significant in Model 4, which incorporates regional characteristics, probably because there are significant geographical differences in compensation for land acquisition, which to some extent weakens the effect of Land Acquisition on individual economic behavior. On the control variables of “Socio-economic Characteristics,” Housing Provident Fund significantly reduced the entrepreneurial decision; the higher the Income Satisfaction, the lower the probability of choosing entrepreneurship. Social Donation significantly facilitated the entrepreneurial decision, and the more proficient the Internet Banking Use, the higher the probability of starting a business. The regression results of “Regional Dummy Variables” show that relative to the central region, then the eastern region has a positive promotion effect, while the western region has no significant effect.

### Endogeneity treatment: instrumental variables approach

Although the endogeneity problem in this paper is not very serious, there is still an estimation bias caused by the omitted variable problem, so this paper adopts the instrumental variable method to deal with it to demonstrate the robustness of the estimation results. We choose “distance from old home to local government” and “nature of household registration at birth” as the instrumental variables. First, if the distance of the respondent’s hometown is far from the government, it is more likely that the hometown is in a rural area, and thus the more likely to have farming experience, so the distance variable is associated with the Farming Experience. The agricultural household registration at birth is associated with the Farming Experience. It is important to note that since Farming Experience and Entrepreneurial Decision are dichotomous variables, if we use a dichotomous variable as an instrumental variable to regress another dichotomous variable, it will cause some identification difficulties. For this reason, this paper uses the Eprobit model in the Extended regression model to deal with endogeneity to examine the robustness of the results of the instrumental variables.

Table 4 shows the regression results of the Eprobit model. The validity test of the Eprobit model is significant at the 10% statistical level, and the residual terms of the two regression equations are correlated in Table 4, indicating that the explanatory variables are indeed endogenous dummy variables and the use of the Eprobit model is justified. The regression of instrumental variables on the explanatory variable farming experience is statistically significant at the 1% level, indicating that the instrumental variables are highly correlated with the explanatory variables. The one-stage *F*-value of 51.51 corresponding to the instrumental variables is higher than the criteria for judging weak instrumental variables in the literature, and thus there is no weak instrumental variable problem. For exogeneity, none of the *p*-values of the overidentification tests were significant, indicating that the original hypothesis of exogeneity of instrumental variables was accepted. The coefficient value of the Farming Experience is positive and significant at the 1% statistical level, which is consistent with the results of the benchmark regression and the conclusions remain robust. However, the coefficient values estimated based on instrumental variables are significantly higher compared to the baseline regression, indicating that if the endogeneity issue is ignored, the positive effect of Farming Experience on urban residents’ Entrepreneurial Decision is underestimated.

**TABLE 4:** Endogenous treatment: instrumental variable method.

	Entrepreneurial decision (1)	Farming experience (2)
Farming experience	0.6453***	
	(0.2215)	
IV (distance from old home to local government)		0.0317***
		(0.0057)
IV (nature of household registration at birth)		0.3346***
		(0.0169)
Control variables	Yes	Yes
_cons	−5.2626***	0.0478***
	(0.7941)	(0.0128)
N Obs.	2,211	2,221

***Indicate significance at the 1% statistical levels, respectively.

### Analysis of heterogeneity

In the previous analysis, we have verified that the Farming Experience has a significant positive effect on entrepreneurship among urban residents. However, the above findings are the average effect examined from the perspective of the whole sample, and further in-depth research is needed for different group heterogeneity. This study groups the study sample according to three dimensions: “Age,” “City Type,” and “Physical Capital,” hoping to make an in-depth extension of this research topic. Table 5 shows that there are significant heterogeneity differences in both “Age” grouping, “City Type” grouping, and “Physical Capital” grouping.

**TABLE 5:** Results of heterogeneity estimation.

	Age division		City type		Physical capital	
	Under 35	Over 35	Provincial capital	Non-capital cities	Low material capital	High material capital
Farming experience	0.213	0.322***	0.106	0.457***	0.117	0.495***
	(0.265)	(0.095)	(0.132)	(0.126)	(0.119)	(0.133)
Control variables	Yes	Yes	Yes	Yes	Yes	Yes
_cons	−2.510***	−2.359***	−4.672***	−***5.510	−***4.809	−***5.028
	(0.741)	(0.421)	(0.896)	(1.222)	(0.927)	(1.202)
Pseudo R2	0.1036	0.1026	0.0726	0.1422	0.0899	0.1089
N Obs.	768	1,868	1,469	1,221	1,434	1,256

*** indicate significance at the 1% statistical levels, respectively.

For “Age” grouping, the paper is divided into “Under 35” and “Over 35,” and it can be seen that farming experience has a significant positive effect on “Over 35”, while it has no significant effect on “Under 35”. Second, in the “City Type” grouping, it is divided into “Provincial capital” and “Non-capital cities”, and the Farming Experience is more significant in the subsample of “Non-capital cities.” For the “Physical Capital” grouping, respondents’ household economic status is used as a variable to measure physical capital. According to the median of family economy, they are divided into “Low-material capital” and “High-material capital.” Table 5 shows that Farming Experience is significantly positive in the “High-material capital” sample but has no significant effect in the “Low-material capital”, indicating that the effect of Farming Experience on Entrepreneurial Decision is more pronounced in the “High-material capital” group. These results validate H2.

## Discussion

Equation 2 is the main regression of the effect of Farming Experience on entrepreneurship. Equation 3 is the effect of Farming Experience on the mediating variable, and Equation 4 is the effect of Farming Experience and the mediating variable on Entrepreneurial Decision, where *X*_*i*_ denotes the explanatory variable Farming Experience, and *M_i_* denotes the mediating variable. According to the test procedure (Wen and Ye, 2014), first, the main effect of Farming Experience on entrepreneurship is tested for significance, i.e., the significance of *c* in Equation 2. Second, the effect of Farming Experience on the mediating variable is estimated, i.e., the coefficient *a* in Equation 3. Third, the effect of Farming Experience on entrepreneurship is examined by including both Farming Experience and the mediating variables in the regression equation, i.e., the coefficients *c*^`^and *b* in Equation 4. If each process is significant, the mediating effect holds. However, if the *a* in Equation 3 and *b*in Equation 4 is not significant, it is necessary to apply the Bootstrap method to directly test H0: ab = 0. If it is significant, the indirect effect still exists, and if it is not significant, the indirect effect does not hold.


(2) $$Y_{i}=c\,X_{i}+\phi$$

(3) $$M_{i}=a\,X_{i}+\beta$$

(4) $$Y_{i}=c^{`}\,X_{i}+b\,M_{i}+\omega$$

The mediating variables chosen in this paper are positive personality traits, which are measured by Optimism and Mutual aid consciousness. For mediating variables, the Optimism variable was designed as “Do you usually have emotional problems”, and the corresponding responses were “No, rarely, sometimes, and often,” and the values were assigned as “1, 2, 3, and 4.” Luthans et al. (2007) classified psychological utility into four dimensions: self-efficacy, optimism, hope, and resilience. And individuals’ usual emotional problems are not only a reflection of optimism and hope, but also an indirect refraction of self-efficacy. The questionnaire design for Mutual aid consciousness is “how often neighbors help each other,” and we assign 0 to “very little, little, and average” and 1 to “a lot, and a lot.” This is a dummy variable for the Mutual aid consciousness.

The results of the test for the mediating effect are shown in Table 6. Column (1) shows the effect of Farming Experience on positive personality traits, and the results show that Farming Experience significantly reduces disillusionment and has a positive effect on the development of a sense of mutuality. Columns (2) to (3) show the effect of Farming Experience and mediating variables on Entrepreneurial Decision, respectively. It can be seen that positive personality traits, consisting of optimism and a sense of mutuality, significantly increase the probability of entrepreneurship, and the values of Farming Experience coefficients tend to decrease compared to the results of the baseline regression, indicating that the mediating variables partially dilute the effect of the explanatory variables. This validates the mechanism of positive personality traits in H3. The above findings suggest that Optimism and Mutual aid consciousness are the transmission mechanisms through which Farming Experience affects labor force entrepreneurship.

**TABLE 6:** Test results of influence mechanism.

Variables	Open personality (1)		Entrepreneurship (2)	Entrepreneurship (3)
	Daily emotional problems	Mutual aid consciousness		
Farming experience	−0.108**	0.355***	0.273***	0.266***
	(0.055)	(0.063)	(0.089)	(0.088)
Optimism			−0.139***	
			(0.036)	
Mutual aid consciousness				0.179**
				(0.073)
Control variables	Yes	Yes	Yes	Yes
Pseudo R2	0.0244	0.0347	0.0983	0.0941
N Obs.	3,091	3,091	2,690	2,690

***, ** indicate significance at the 1, and 5% statistical levels, respectively.

## Conclusion

This paper examines the impact of farming experience on urban residents’ entrepreneurial decisions based on 2016 CLDS data. The estimation results show that having farming experience can play a significant role in promoting urban residents’ entrepreneurial decisions compared to those without farming experience. To verify the robustness of the estimation results, this paper applies the instrumental variables method to test the results, and the results remain significant. The analysis of potential mechanisms of action revealed that the farming experience indirectly promotes entrepreneurial decisions through the mediating role of promoting positive personality traits such as optimism and a sense of mutuality. The results of the subsample discussion show that the effect of farming experience on the entrepreneurial decision is more significant in the middle-aged group, in non-capital cities, and in groups with high-physical capital.

Farming experience has an impact on economic behavior, which has implications for understanding residents’ entrepreneurial decisions in terms of micro-individual characteristics. We argue that positive personality traits such as optimism and mutual aid can be used as theoretical mechanisms to promote entrepreneurial decision-making through farming experiences, thus fostering positive personality traits that can help complement formal social institutions to promote individual entrepreneurial intentions. It is undeniable that entrepreneurship is an important factor in promoting economic development, as it not only contributes to solving social employment problems, but is also important in promoting labor demand and achieving fuller and higher quality employment. Distinguishing from previous studies that have focused on macroeconomic aspects to enhance the scale of urban entrepreneurship, the findings of this study imply the importance of micro-individual characteristics to influence entrepreneurial decisions. Based on the main conclusions above, the following policy recommendations are proposed:

Education and cultivation of residents’ positive personality traits should be strengthened to enhance their ability to cope with risks, especially youth groups should be the focus of cultivation, and the construction of public cultural services in non-capital cities should be strengthened. When formulating public policies on entrepreneurship, the specificity of farming experience in the process of urbanization should be taken into account, and the application of farming skills in urban life should be brought into play. Broadening residents’, the third is to broaden the channels for residents to acquire material capital and promote the accumulation of material capital to enhance the material security of residents’ entrepreneurial decisions.

This paper studies the relationship between agricultural experience and people’s entrepreneurship. However, as entrepreneurship is a part of venture capital, it is planned to carry out the correlation between agricultural experience and venture capital in future research, including the similarities and differences between fund investment, stock investment, and bank time deposit. At the same time, this paper focuses on the indirect effect of personality characteristics. The follow-up study will further combine the relationship between personality characteristics and social and economic activities to explore the relationship between personality characteristics and new forms of employment. Of course, obtaining continuous dynamic tracking data is also the focus of this paper in the future.

## Data availability statement

The original contributions presented in this study are included in the article/supplementary material, further inquiries can be directed to the corresponding author.

## Author contributions

DZ carried out the study and analyzed the data. DZ and LL drafted the manuscript and were responsible for writing the literary and revising the language. LL provided the guidance for revising the manuscript. Both authors read and approved the final manuscript.
